# BOEC–Exo Addition Promotes In Vitro Maturation of Bovine Oocyte and Enhances the Developmental Competence of Early Embryos

**DOI:** 10.3390/ani12040424

**Published:** 2022-02-10

**Authors:** Yiran Wei, Muhammad Idrees, Tabinda Sidrat, Myeondon Joo, Lianguang Xu, Jonghyeok Ko, Ilkeun Kong

**Affiliations:** 1 Department of Animal Science, Division of Applied Life Science, Institute of Agriculture and Life Science (IALS), Gyeongsang National University, Jinju, Gyeongnam 52828, Korea; weiyiran1230@gmail.com (Y.W.); idrees1600@gmail.com (M.I.); tabindasidrat06@gmail.com (T.S.); jmd1441@gmail.com (M.J.); xulianguang428@gmail.com (L.X.); 2 Institute of Agriculture and Life Science, Gyeongsang National University, Jinju, Gyeongnam 52828, Korea; 3 The King Kong Corp. Ltd., Gyeongsang National University, Jinju, Gyeongnam 52828, Korea; jonghyeokk@gmail.com

**Keywords:** exosome, bovine oocyte, in vitro maturation, gap junction, cumulus cell culture, embryo development, embryo implantation

## Abstract

**Simple Summary:**

The results of the present study proved that the addition of bovine oviductal epithelial cell derived exosomes (BOEC–Exo) to the in vitro maturation (IVM) media improved the bovine oocyte maturation and early embryo development. The addition of BOEC–Exo not only significantly enhanced the polar body exclusion, but also enhanced the expression of connexins in cumulus oocyte complexes (COCs). Likewise, the reactive oxygen species (ROS) level, protein expressions of SIRT-1, and mitochondrial membrane potential (ΔΨm) also suggested that BOEC–Exo addition to IVM media is highly beneficial for in vitro bovine oocyte maturation. Furthermore, BOEC–Exo treatment to the primary cultured bovine cumulus cells significantly attenuated apoptosis, which also showed its positive influence on the COCs. Moreover, oocytes that were matured in the presence of BOEC–Exo led to the production of a significantly higher quantity and quality of day-8 blastocysts. Additionally, the BOEC–Exo treated blastocysts had a higher implantation potential when compared with the control. Our results suggest that the addition of BOEC–Exo to IVM media significantly enhanced the percentage of oocytes maturation and improved the embryo quantity and quality.

**Abstract:**

Exosomes are nano-sized vesicles with abundant nucleic acids, proteins, lipids, and other regulatory molecules. The aim of this study was to examine the effect of BOEC–Exo on bovine in vitro oocyte maturation and in vitro embryo development. We found that a 3% Exo supplementation to IVM media significantly enhanced the oocyte maturation and reduced the accumulation of ROS in MII-stage bovine oocytes. Oocyte maturation related genes (GDF9 and CPEB1) also confirmed that 3% Exo treatment to oocytes significantly (*p* < 0.05) enhanced the oocyte maturation. Next, we cultured bovine cumulus cells and assessed the effects of 3% Exo, which showed a reduced level of apoptotic proteins (caspase-3 and p-NF-κB protein expressions). Furthermore, we examined the gap junction (CX43 and CX37) and cumulus cells expansion related genes (HAS2, PTX3, and GREM1) in cumulus–oocyte complexes (COCs), and all those genes showed significantly (*p* < 0.05) higher expressions in 3% Exo-treated COCs as compared with the control group. Moreover, peroxisome proliferator-activated receptors (PPARs) and lipid metabolism-related genes (CPT1 and FABP3) were also analyzed in both the control and 3% Exo groups and the results showed significant (*p* < 0.05) enhancement in the lipid metabolism. Finally, the oocytes matured in the presence of 3% Exo showed a significantly higher rate of embryo development and better implantation potential. Finally, we concluded that Exo positively influenced bovine oocyte in vitro maturation and improved the early embryo’s developmental competence.

## 1. Introduction

The development of in vitro produced (IVP) embryos is important for cattle production. For bovine IVP embryos, COCs are collected from abattoir-acquired ovaries and they complete the oocyte meiotic maturation in vitro (IVM). IVM oocytes have the capacity to be fertilized and develop into embryos, but the success rate is far lower than the in vivo-matured oocytes [1]. IVM media have been extensively studied, but there is still no consensus regarding the optimal method that satisfies the needs of oocyte maturation [2]. Several studies demonstrated that the developmental competency of the embryo is highly influenced by the oocyte maturation medium [3,4]. According to prior research, appropriate medium composition and culture conditions are critical for oocyte IVM success [4,5].

To accomplish complete oocyte maturation, both nuclear and cytoplasmic maturation are required, and inadequate maturation has been linked to low embryo developmental potential [6]. Tissue culture Medium 199 (TCM199) supplemented with estradiol-17β, gonadotropins, and 10% serum has traditionally been used to enhance in vitro oocyte maturation [7]. TCM199 is a commercial medium used for in vitro bovine oocyte maturation, but it is not specifically designed for oocyte maturation [8]. TCM199 lacks the conditions provided by oviductal fluid, which contains an abundance of growth factors, hormones, immunomodulatory ingredients, and extracellular exosomes, all of which are important in reproductive and developmental processes [9,10,11]. Exosomes are membrane-coated extracellular vesicles with a diameter of 30–200 nm, secreted into the extracellular environment by somatic cells [12,13,14]. Exosomes comprise a phospholipid bilayer membrane and carry proteins, lipids, mRNAs, microRNAs, DNA fragments, and several unknown compounds [15]. Our team recently published a study that found that adding bovine epithelial cell-derived exosomes (BOEC–Exo) to the developing embryo increased metabolic flux and supported in vitro bovine embryonic development up to the blastocyst stage [15]. Another study investigated the effects of exosomes on cloned bovine embryo development [16], but none have examined its impact on oocyte maturation. 

Exosomes can act not only as biomarkers, but also as targeted drug-delivery systems, and thus hold promise for an advanced comprehension of reproductive diseases [17]. However, the precise mechanisms of action of exosomes in oocyte maturation have not been elucidated yet. In this study, we added BOEC–Exo to the oocyte maturation medium and analyzed its effects on bovine oocyte nuclear and cytoplasmic maturation. We examined the transcription levels of oocyte maturation-related genes in oocytes and cultured cumulus cells. Furthermore, we also examined reactive oxygen species (ROS), and lipid contents in the Exo-treated oocytes. Moreover, the oocytes matured in the presence of Exo were further fertilized and cultured in in vitro embryo development (IVP) media and we assessed the developmental rates and implantation potential of the resulting embryos. 

## 2. Materials and Methods

All reagents mentioned in materials and methods were purchased from Sigma-Aldrich (St. Louis, MO, USA) unless otherwise specified. 

### 2.1. Experimental Design

Experiment 1: Immature bovine COCs were aspirated from abattoir ovaries and matured in the control (IVM media only) and Exo (IVM + 3% Exo) groups. The denuded matured oocytes were collected for ROS assay, DAPI staining, and JC-1 staining. For gene expression analysis, qRT-PCR was used to analyze the gene expressions in the control and 3% Exo-treated mature oocytes. Matured COCs (with 3–4 layers of cumulus cells) from both the groups were used to analyze the gene expressions related to cumulus cell expansion and gap junctions. 

Experiment 2: Primary bovine cumulus cells separated from immature COCs were cultured for 24 h and then treated with control and 3% Exo for the next 22 h. The samples were collected for gene expression analysis via qRT-PCR. Furthermore, the apoptosis in cultured cumulus cells was analyzed via immunofluorescence (Caspase 3 and p-NF-κB).

Experiment 3: The control and 3% Exo-treated matured oocytes were in vitro fertilized and cultured in the IVC medium. At day 8, blastocyst developmental rates were assessed and used for immunofluorescence (PPARδ) and implantation assay.

### 2.2. Aspiration and Collection of Oocytes

Bovine ovaries were collected from a local slaughterhouse in Gimhae, Republic of Korea, and transported to the lab within 1.5 h in physiological saline (0.9 percent NaCl) and at 37.5 °C [18]. To remove any blood contamination, the ovaries were washed in fresh Dulbecco’s phosphate-buffered saline (D-PBS) following arrival. COCs were collected from follicles ranging in size from 2 to 8 mm using an 18 G needle connected to a vacuum pump. TL-HEPES medium (2.383 g/L HEPES, 6.662 g/L sodium chloride, 1 μL/mL phenol red, 0.168 g/L sodium bicarbonate, 0.85 g/L sodium lactate, 0.101 g/L calcium chloride, 0.238 g/L potassium chloride, 0.040 g/L sodium biphosphate, 0.101 g/L magnesium chloride, 100 IU/mL penicillin, and 0.1 mg/mL streptomycin) was used for the aspiration of the COCs. A stereomicroscope (SZ6045, Olympus, Tokyo, Japan) was used to select oocytes with homogenous cytoplasm and at least three layers of compact cumulus cells.

### 2.3. Bovine Primary Cumulus Cells Culture

The cumulus cells were collected from 3–6 mm immature bovine COCs and culture as previously described [19]. Briefly, the cumulus cells were cultured in collagen-coated 24-well plates with 1.25 × 10^5^ concentration per well. The cells’ viability was analyzed by trypan blue, and the cells were counted with a cell cytometer. For the cell culture, a serum-free media (α-MEM media supplemented with bovine serum albumin (BSA, catalog no. A6003, sigma-Aldrich, St. Louis, MO, USA, 0.1%), penicillin (100 IU), and streptomycin (0.1 mg/mL), sodium selenite (4 ng/mL), HEPES (20 mM), transferrin (5 μg/mL), sodium bicarbonate (0.084%), insulin (10 ng/mL), androstenedione (2 μM), L-glutamine (2 mM), nonessential amino acids (1 mM)) was used. 

### 2.4. In Vitro Maturation of Ooocytes

For the in vitro maturation of the immature oocytes, we used the protocol previously mentioned [2,6,18]. Briefly, the ovary-aspirated COCs were washed and cultured in IVM medium (TCM199 supplemented with 10% (*v*/*v*) fetal bovine serum, 10 μg/mL EGF, 0.2 mM sodium pyruvate, 10 μg/mL follicle-stimulating hormone, 1 μg/mL estradiol-17β, and 0.6 mM cysteine). After that the COCs in 4-well plates (around 40 oocytes per well) were incubated at 38.5 °C in a 5% CO_2_ humidified atmosphere for 22 h.

### 2.5. DAPI Staining for Oocyte Meiotic Maturation Assessment

To detect the nuclear maturation, the in vitro matured COCs (22 h later) were collected, and cumulus cells were removed by 0.1% hyaluronidase treatment and gentle pipetting. A stereomicroscope (SZ6045, Olympus, Tokyo, Japan) was used to analyze the first polar body extrusion. The oocytes were treated with Triton X-100 (0.5%) for at least 10 min for permeabilization, and then stained with 4′,6-diamidino-2-phenylindole (DAPI catalog no. 62248, Thermo Fisher Scientific, Waltham, MA, USA) for 5 min. The extrusion of the polar body was detected under an epifluorescence microscope (Olympus IX71, Olympus, Tokyo, Japan). The oocytes were classified as mature (MII) or immature oocytes based on the morphology of the nuclear material. 

### 2.6. In Vitro Fertilization

For in vitro fertilization, we again used our lab established protocol [6,18]. Briefly, the in vitro-matured COCs were co-cultured with frozen–thawed semen from a Hanwoo bull previously tested for IVF. Water at a temperature of 38 °C was used to thaw the frozen sperm straw for 30 sec, and DPBS was used to wash the sperm. After that, sperm pellets were obtained by centrifugation (750× g) for 5 min. To facilitate capacitation, the pelleted semen was resuspended in 500 μL of heparin (20 mg/mL) prepared in in vitro fertilization (IVF) medium (Tyrode lactate solution supplemented with 6 mg/mL BSA, 22 mg/mL sodium pyruvate, 0.1 mg/mL streptomycin, and 100 IU/mL penicillin) and incubated at 38.5 °C in a humidified atmosphere of 5% CO_2_ for 15 min. The spermatozoa were then diluted to a final concentration of 1 × 10^6^ spermatozoa per milliliter. The 0.5 mL of IVF was added to almost 40 COCs per well in 4-well dishes and incubated under a humidified atmosphere of 5% CO_2_ in air at 38.5 °C for 18–20 h.

### 2.7. In Vitro Culture

For the embryo culture, the presumed zygotes were pipetted, and the cumulus cells were removed. The zygotes were then cultured for 8 days considering IVF as day 0 [6]. Synthetic oviductal fluid (SOF) supplemented with 5 ng/mL sodium selenite, 5 μg/mL transferrin, 5 μg/mL insulin (Sigma-Aldrich cat. #11074547001), 100 ng/mL EGF, and 4 mg/mL fatty acid-free BSA was used as the culturing media and 38.5 °C and 5% CO_2_ were the incubator conditions. 

### 2.8. Supplementation of BOEC–Exo

The procedure of the isolation of bovine oviductal epithelial cell-derived exosomes was previously described in detail [15]. Briefly, bovine oviducts were collected from the local slaughterhouse and transported to the lab within 2 h in ice cold DPBS. The oviducts were then washed in cold D-PBS containing 100 IU/mL penicillin and 100 g/mL streptomycin. The excess fat and tissue around the oviducts were manually removed. Next, the clean oviducts were cut into pieces and placed in HEPES-buffered Medium 199 supplemented with 100 IU/mL penicillin and 100 μg/mL streptomycin. The BOECs were scraped and squeezed out from the oviducts. These BOECs were then centrifuged twice at 550× *g* and 25 °C for 5 min to remove the blood cells and fats. To encourage exosome secretion, the cells were cultured for 24 h in Medium 199 supplemented with 10% fetal calf serum, 100 g/mL streptomycin, and 100 U/mL penicillin. After 24 h, the cells creating Exo were centrifuged again at 550× *g* and 25 °C for 5 min. We discarded the liquid supernatant, and the cells was suspended in DMEM/Ham’s F12 medium (catalog no. 10565-018, Gibco BRL, Paisley, UK) supplemented with 5% fetal calf serum, 10 ng/mL epidermal growth factor, 5 μg/mL insulin, 5 μg/mL transferrin, 10 mM glutathione, 50 nM trans-retinoic acid, 2.5 mg/mL amphotericin B, and 100 μg/mL gentamycin. The cells were cultured for 7 days with a concentration of 3 × 10^6^ cells/mL, and the incubator conditions were 38.5 °C and 5% CO_2_. The Exo were finally collected and purified by centrifugation at several different speeds and kept in cold PBS. After this, they were characterized via nano-tracking analysis to confirm the size (80–150 nm) and distribution of the Exo (3 × 10^8^ particles per mL). The Exo concentration was described in our previous work, in which we found that the addition of 3% Exo to embryo development media highly improved the blastocysts development and hatching percentage. Therefore, we selected 3% Exo and used it to evaluate in vitro oocyte maturation.

### 2.9. RNA Extraction, cDNA Synthesis, and Quantitative Reverse Transcription PCR (q-RT-PCR) Analysis

All of the RNA was extracted using a PicoPure^TM^ RNA isolation kit (Thermo Fisher, Foster City, CA, USA) following the manufacturer’s protocol, 30 COCs per group. An iScript^TM^ cDNA synthesis kit (Bio-Rad Laboratories, Hercules, CA, USA) was used to reverse-transcribe mRNA into first-strand cDNA. The expressions of 16 different genes were selected to describe the differences between the control and Exo groups as regards the metabolism, gap junction communication, mitochondrial activity, and apoptosis in oocyte and cumulus cells. qRT-PCR was performed in three independent experiments with four replicates by using a CFX96 instrument (Bio-Rad Laboratories, Hercules, CA, USA). The cycling conditions were as follows: 94 °C for 5 min, followed by 40 cycles of 94 °C for 30 s, 58 °C for 30 s, and 72 °C for 30 s. The mRNA expressions of the aforementioned genes were quantified by ΔΔ Ct method using GAPDH as the reference gene [6,18,20]. The primer sequences are listed in Table 1.

### 2.10. Nile Red Staining

The cumulus cells were removed from the matured oocytes, fixed in 4% formaldehyde, and stored at 4 °C for a minimum of 24 h. On the day of staining, the oocytes were washed thrice in Polyvinyl Pyruvate (0.3%)-PBS, and then were stained with 10 μΜ Nile red solution (Sigma-Aldrich cat. # 72485) at room temperature for 20 min. After staining, the oocytes were transferred onto glass slides and covered with coverslips after being washed three times in PVP-PBS. A confocal laser-scanning Olympus Fluoview FV1000 microscope (Olympus, Tokyo, Japan) was used to take fluorescence pictures, which were taken with an emission of 485 nm as Nile red dye. ImageJ software (version 1.50, National Institutes of Health, Bethesda, MD, USA; https://imagej.nih.gov/ij, accessed on 22 November 2021) was used to measure the intensities of fluorescence. 

### 2.11. ROS Assay

For ROS quantification in the control and Exo-treated MII stage oocytes, 2′,7′-Dichlorodihydrofluorescein diacetate (H_2_DCFDA) (Sigma-Aldrich cat. #D6883) was used. Briefly, the control and Exo-treated oocytes were incubated for 30 min in a PBS-PVP solution containing 10 M/mL DCHFDA at 37 °C in 5% CO_2_. After that, the oocytes were washed three times with PBS-PVP solution and analyzed with a 490 nm excitation and 525 nm emission filter using an epifluorescence microscope (IX71, Olympus, Tokyo, Japan).

### 2.12. Immunofluorescence

To detect the protein expression, we used immunofluorescence staining as previously described [19]. Briefly, in vitro matured MII stage bovine oocytes (30 per group) or day-8 bovine embryos (15 per group) or cumulus cells were fixed in 4% (*v*/*v*) paraformaldehyde and stored at 4 °C until further experiment. On the experiment day, the samples were taken into 48-well dishes (all the operations of cumulus cells were carried out in collagen-coated 24-well plates) and washed three times in PBS-PVP (0.3% PVP 1× PBS) for 10 min. The samples were treated with proteinase K solution (catalog no. 158918, Qiagen, Hilden, NW, Germany) for 5 min to permeabilize for antigen retrieval. Next, the samples were washed thrice and incubated in blocking buffer (bovine serum and 0.1% Triton X-100 in DPBS) at room temperature (RT) for 1 h. After blocking, the primary antibody was applied, and the samples were kept on a rotator overnight. On the next day, after washing the samples three times, they were incubated with secondary antibodies (TRITC and FITC, Santa Cruz Biotechnology, Dallas, TX, USA) for 90 min at RT. After the secondary antibodies, the samples were washed again and treated with DAPI for 5 min. Following the last three washes, the stained samples were placed on a slide and covered with a slide cover. A laser-scanning confocal microscope (Fluoview FV 1000, Olympus, Tokyo, Japan) was used to take images. The ImageJ program was used to measure the relative integrated density of the signals.

### 2.13. Antibodies

The following antibodies were used in this study: caspase-3 (catalogue no. sc-1225, Santa Cruz Biotechnology, Dallas, TX, USA), p-NF-kB (catalogue no. sc-271908; Santa Cruz Biotechnology, Dallas, TX, USA), SIRT1 (catalogue no. sc-15404, Santa Cruz Biotechnology, Dallas, TX, USA), and PPARδ (catalogue no. LS-C437498 LS Bioscience Seattle, WA, USA). 

### 2.14. JC-1 Staining

The JC-1 (5,5’,6,6’-tetrachloro-1,1’,3,3’-tetraethylbenzimidazolylcarbocyanine iodide) staining was carried out according to the protocol previously mentioned [21]. Briefly, MII stage oocytes in the control and Exo treatment groups were stained with 10 mg/mL JC-1 dye (catalogue no. T3168, Invitrogen, Carlsbad, CA, USA) (diluted in PBS-PVP) at 38.5 °C for 1 h in a 5% CO_2_ incubator. After that, the oocytes were washed with PBS-PVP solution three times. The oocytes were then assembled on slides, covered with slide covers, and analyzed under a laser scanning microscope (Fluoview FV1000, Olympus, Tokyo, Japan). 

### 2.15. Invasion Assay

For the invasion assay, the same protocol as has been previously mentioned in published articles was used [6]. Briefly, day-8 hatched bovine blastocysts were placed in 24-well tissue culture plates (Corning Inc. Life Sciences Corning, New York, NY, USA). Before commencing, the filter surface was coated with Matrigel (20 mg per filter: Discovery Labware Inc. Billerica, MA, USA) and then incubated at 37 °C for 2 h. No more than 3 blastocysts were placed on a filter with the same medium as was used for embryo development. The embryos were kept in an incubator under a 5% CO_2_ and 38.5 °C for 72 h. Next, the culture medium was changed from IVC1 (in IVC1, fetal bovine serum (FBS) was used) to IVC2 (in IVC2, KSR (knockout serum replacement (KSR) Gibco cat # 10828010) was used) and refreshed every 24 h [22]. On Day 10 of the culturing, the samples were washed 3 times in PBS/PVP, then fixed in 4% (*v*/*v*) paraformaldehyde prepared in 1 M DPBS. For invasion evaluation, the samples were washed in PBS/PVP and stained with DAPI for 5 min, and then washed 3 times to remove excess stain. The picture of the invasion area was taken with an Olympus IX71 microscope and analyzed by ImageJ software.

### 2.16. Statistical Analysis

Statistical analysis was performed using GraphPad PRISM v 6.0 software (GraphPad 6 Software Inc. Inc., La Jolla, CA, USA). The analyses were performed using a *t*-test, followed by a paired comparison test. The images of the ROS assay, immunofluorescence, JC-1 staining, Nile red, and invasion assay were used to analyze the signal’s intensity (and invasion area) via ImageJ software. The density values of the data were expressed as the mean ± SEM of three independent experiments. Significance: * = *p* < 0.05.

## 3. Results

### 3.1. Developmental Competence of In Vitro Matured Oocytes in the Presence of BOEC–Exo

**Figure 1:**
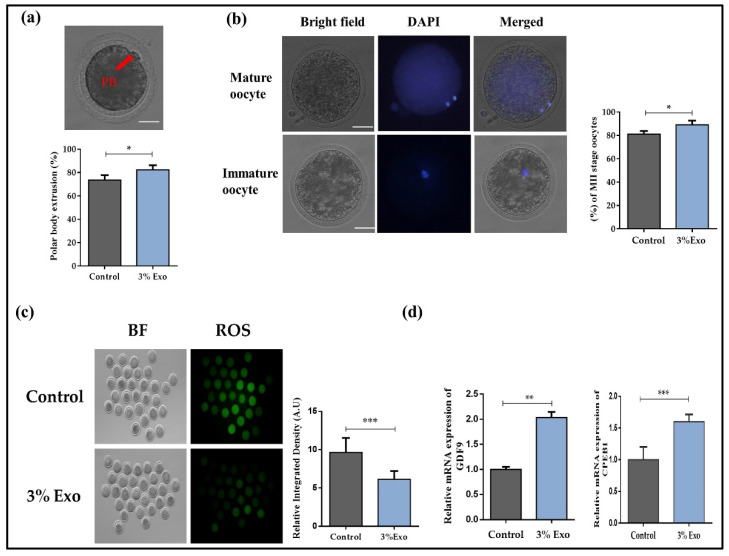
The effect of Exo on oocyte maturation. (**a**) First polar body (PB) in matured oocyte under microscope and percentage of oocytes with polar body extrusion. Original magnification 100×; (**b**) The appearance of MII (Metaphase II, matured oocytes) stage after DAPI staining and percent-age of MII stage oocytes. Original magnification 100×; (**c**) Oocytes after 22 h maturation in control and 3% Exo group stained by DCHDFA and generation of ROS level. Original magnification 40×; (**d**) relative mRNA level of GDF9 and CPEB1 in control and 3% Exo groups after 22 h of maturation. With a total of 30 oocytes per group and the experiment was repeated for 6 times. Data in the bar graphs represent the means ± SEM from at least three independent sets of experiments. * *p* < 0.05, ** *p* < 0.001, and *** *p* < 0.0001.

To initiate our experiment, we first analyzed the degree of oocyte maturation in the control and 3% Exo-added media. The 3% Exo-addition group showed significantly enhanced first polar body extrusion (Figure 1a, *p* < 0.05). To verify high maturation percentage, we carried out DAPI staining and found higher meiotic maturation of oocytes in the presence of Exo (Figure 1b, *p* < 0.05). After that, we analyzed the ROS levels in both the control and 3% Exo group, and the results revealed a decreased ROS level in the Exo group (Figure 1c, *p* < 0.05). To examine the effects of 3% Exo on the oocyte’s mRNAs, we performed a qRT-PCR and analyzed the expressions of genes related to developmental competency, such as GDF9 (growth differentiation factor 9), and CPEB1 (cytoplasmic polyadenylation element binding protein 1). The results showed a significant enhancement of the expressions of GDF9 and CPEB1 genes in the Exo group (Figure 1d, *p* < 0.05). The above results showed that addition of 3% Exo to the IVM medium significantly improved the meiotic maturation of bovine oocytes.

### 3.2. Effect of BOEC–Exo on Gap Junction Communication and Cumulus Cell Health

**Figure 2:**
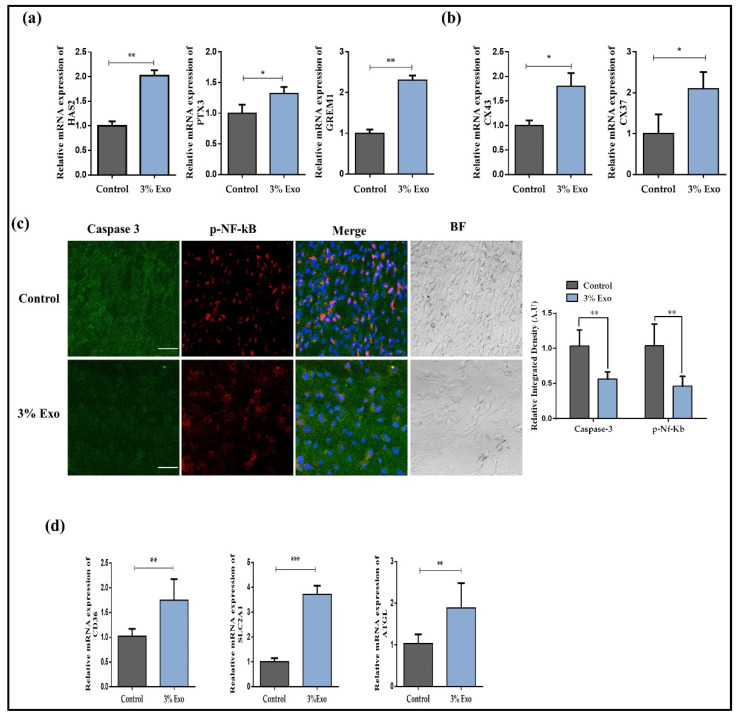
Effect of Exo on oocyte surrounding cumulus cells. (**a**) Relative mRNA level of HAS2, PTX3, and GREM1 in cumulus-oocyte complexes. (**b**) Effect of Exo on junctional communication. The relative mRNA expressions of CX43 and CX37 in mature cumulus oocyte complexes (30 per group); (**c**) immunofluorescent images of caspase-3 and p-NF-κB in cultured cumulus cells and integrated optical density in both control and 3% Exo groups. Original magnification 100×; (**d**) Relative mRNA expression of CD36, SLC2A1, and ATGL in control and 3% Exo-treated cultured cumulus cells. Data in the bar graphs represent the means ± SEM from three independent sets of experiments. * *p* < 0.05, ** *p* < 0.001, and *** *p* < 0.0001.

Cumulus cell expansion is necessary for oocyte meiotic maturation and for acquiring developmental competence [23]. The effects of 3% Exo on cumulus cell expansion were investigated by looking into the genes involved in cumulus cell expansion in COCs. The results showed that HAS2 (Hyaluronan Synthase 2), PTX3 (Pentraxin 3), and GREM1 (Gremlin 1) genes were significantly enhanced in the Exo group (Figure 2a, *p* < 0.05). Gap junctions between cumulus cells and the oocyte play a significant role in meiotic maturation of oocyte. So, we analyzed CX43 and CX37 mRNAs expressions (Figure 2b, *p* < 0.05) [24]. We found that a 3% Exo addition to the IVM media significantly enhanced the expression of both genes. Apoptosis in the oocytes surrounding cumulus cells highly reduce the oocyte meiotic maturation. We cultured bovine cumulus cells and treated them with 3% Exo for 22 h (due to the maturation time of bovine oocytes). After that we analyzed the expressions of apoptosis-related proteins via immunofluorescence microscopy (Figure 2c, *p* < 0.05). The results showed that the protein level of caspase-3 was much lower in the 3% Exo-added group compared to the control, while p-NF-κB also displayed lower nuclear localization in the 3% Exo group. Fatty acid metabolism in cumulus cells highly influences oocyte meiotic maturation [25], so we assessed the gene expressions of CD36 (cluster of differentiation 36), SLC2A1 (solute carrier family 2 member 1), and AGTL (adipose triglyceride lipase) in cumulus cells (Figure 2d, *p* < 0.05). The results showed significant enhancements in the expressions of all these genes in the 3% Exo group. All the above results show that the addition of 3% Exo significantly improved the medium for in vitro maturation of bovine oocytes. 

### 3.3. BOEC–Exo Addition Improves the Glucose Metabolism and Mitochondrial Activity in In Vitro Mature Oocytes

**Figure 3:**
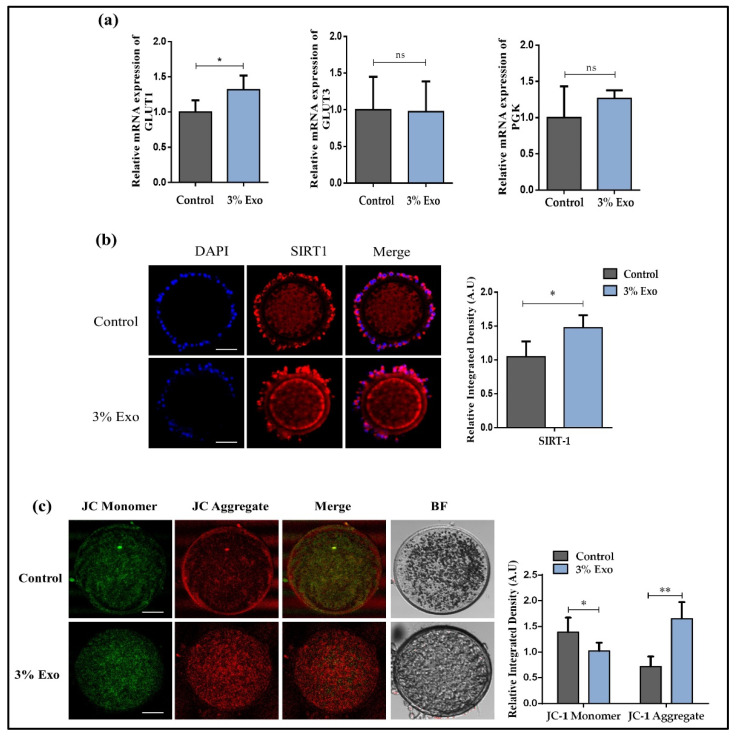
Exo addition improves the glucose metabolism and mitochondrial activity during the in vitro maturation of COCs. (**a**) Relative mRNA level of GLUT1, GLUT3, and PGK in control and 3% Exo COCs; (**b**) immunofluorescence of SIRT1 expression in COCs and integrated optical density of SIRT1. Original magnification 100×; (**c**) representative images of JC-1 staining of control and 3% Exo-treated MII stage oocytes; 30 COCs per group. Original magnification 100×. Data in the bar graphs represent the means ± SEM from three independent sets of experiments. * *p* < 0.05, ** *p* < 0.001.

To further evaluate the effects of adding 3% Exo to IVM, we analyzed the glucose metabolism and mitochondrial activity of matured oocytes. We found that the gene expression of glucose transporter 1 (GLUT1) was significantly enhanced in the 3% Exo group, as compared with the control group, but there were no significant changes in the glucose transporter 3 (GLUT 3) (*p* < 0.05) and PGK1 (phosphoglycerate kinase 1) (*p* < 0.05) genes (Figure 3a). Sirtuin 1 (SIRT1) also plays an essential role in lipid metabolism, and we analyzed its protein expression in MII-stage oocytes. We found that the protein expression of SIRT1 was significantly higher in the 3% Exo group as compared with the control (Figure 3b, *p* < 0.05). To investigate the influence of 3% Exo on oocyte mitochondrial health and mitochondrial membrane potential (ΔΨm), we stained the mature oocytes from both groups with JC-1 dye. We found that the JC aggregate was much higher in the 3% Exo group as compared with the control group (Figure 3c, *p* < 0.05). These results corroborated the previous finding that Exo improved the metabolism and mitochondrial health in MII-stage bovine oocytes [15,26,27]. 

### 3.4. BOEC–Exo Addition Affects Lipid Level in MII-Stage Bovine Oocytes, Improving Blastocysts’ Development Rate and Implantation Potential

**Figure 4:**
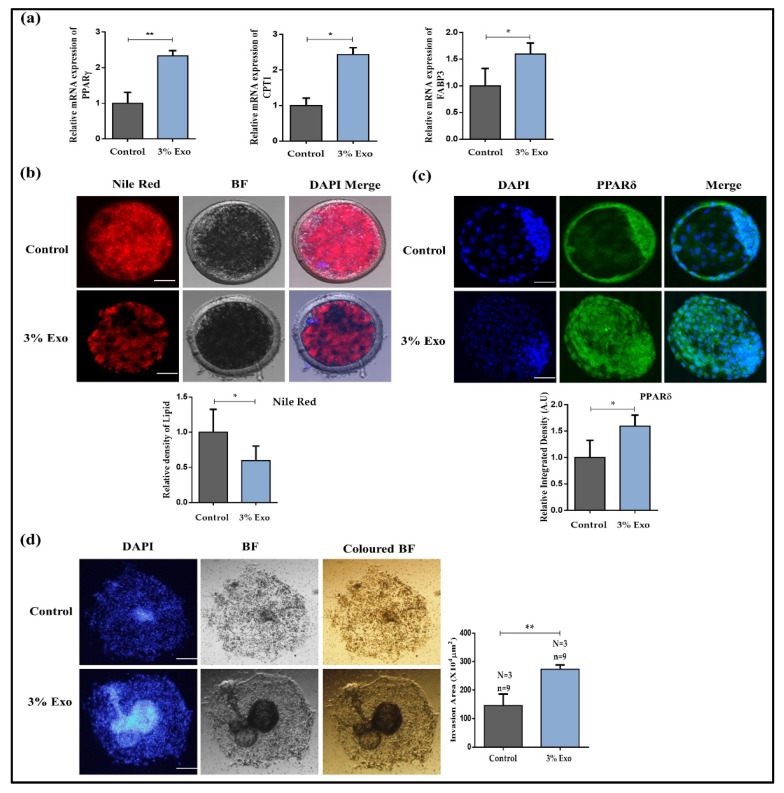
Effect of Exo on oocytes and blastocysts lipid metabolism. (**a**) Relative mRNA expression of PPARγ, CPT1, and FABP3 in control and 3% Exo groups; (**b**) relative density of lipid was examined in both control and 3% Exo-treated oocytes (30 per group), via Nile red dye. Original magnification 100×. (**c**) Immunofluorescence of PPARδ in day-8 blastocysts and integrated optical density in both control and 3% Exo groups (15 per groups). Original magnification 40×. (**d**) The effect of Exo on blastocyst implantation potential was determined. Colored BF (bright field), BF, and DAPI images Scheme 3. Exo groups. Original magnification 40×. Data in the bar graphs represent the means ± SEM from three independent sets of experiments. * *p* < 0.05, ** *p* < 0.001.

Excessive accumulation of lipid significantly compromises oocyte maturation and development of embryos [28,29]. We examined the expressions of lipid-related genes and identified that the levels of PPARγ (proliferator-activated receptor gamma), CPT1 (carnitine palmitoyltransferase 1), and FABP3 (fatty acid binding protein 3) were significantly high in the 3% Exo group (Figure 4a, *p* < 0.05). After that, we directly examined the lipid levels via Nile red staining, and the results clearly showed a significant reduction in the lipid levels in the 3% Exo group (Figure 4b, *p* < 0.05). Likewise, the oocyte matured in the presence of 3% Exo showed a significantly higher blastocysts development rate (36.1%) as compared with control group (28.2%) (Table 2). 

Recently, a study linked proliferator-activated receptor delta (PPARδ) expression with embryo developmental competence, and we found higher expression of PPARδ in the 3% Exo group as compared with the control group (Figure 4c, *p* < 0.05) [21,30]. Furthermore, the considerable difference in blastocyst hatching rates between the two groups corroborates our previous findings. Moreover, we analyzed day-8 bovine embryo implantation potential via an invasion assay, and we found that the area of invasion was significantly higher in the 3% Exo group (Figure 4d, *p* < 0.05).

## 4. Discussion

This study was designed to explore the effects of BOEC–Exo on in vitro bovine oocytes maturation and early embryo developmental competence. We found that the addition of Exo to IVM medium significantly improved oocyte maturation, which plays a central role in the IVP system. Furthermore, the Exo-treated oocytes had more controlled ROS levels and a lower lipid concentration compared with the control group. Moreover, the 3% Exo-treated oocytes showed significant enhancements in embryo developmental rate and had a higher implantation potential. 

The oviduct of a mammal is a highly specialized structure that plays a critical role in the reproductive process. The mammalian oviduct does more than just carry ovulated ova, spermatozoa, and growing embryos between the ovary and the uterus. The functioning oviduct is a living organ that regulates and maintains a fluid-filled environment. Tubal (oviductal) fluid is a good habitat for sperm captivity, oocyte fertilization, and early embryo development [11]. Several studies have found that bovine epithelial cell-derived exosomes play an important role in in vitro embryo development [15,31]. In our study, we found that the presence of Exo in IVM media enhanced the expression of several oocyte developmental competency-related genes (Figure 1) [32,33,34]. Previously, several studies stated that exosomes had a significant effect on reducing accumulated oxidative stress [35,36]; thus, we examined the effects of Exo on the ROS level during bovine in vitro maturation, and the results showed a significant reduction in ROS levels in MII-stage oocytes. 

Exosomes are identified by the presence of biochemically relevant contents that are comparable to but distinct from those of the donor cell, demonstrating that exosome cargo loading is a highly controlled process [37]. The presence of nucleic acids in exosome cargo is the most striking trait. Exosomes contain a variety of RNAs with a concentration of long and short RNA, and mRNAs, which has been shown to be effectively translated in donor cells. Exosomes play an important role in intercellular communication and have been isolated from a wide variety of biofluids and tissues, including plasma, liver, uterine, semen, embryo, etc. [17]. The role of exosomes in intercellular communication comprises mediating the exchange of substances between cells, thereby changing the biological properties of the recipient cells [26,37,38,39]. A previous study identified that exosomes transfer mRNAs to recipient cells and recipient cells can translate those mRNAs [15]. Furthermore, microRNAs of exosomes also regulate the gene expressions of the recipient cells [15]. Exosomes are well-known for their role in cell-to-cell communication, and we hypothesized that BOEC–Exo may transfer mRNA, protein, and other nutrients to the oocyte during meiotic resumption [13,15,26]. In our study, we found high gap junctional communication between cumulus cells and oocytes, which showed a quick resumption of meiotic maturation in the Exo-added group compared to the control group (Figure 2) [19]. Several studies have demonstrated the importance of cumulus cells for oocyte maturation and the acquirement of complete embryonic developmental competence [23,40]. By transferring the molecules to the oocyte via gap junctions, cumulus cells play an important role in resuming the oocyte meiotic arrest [41]. Reduction in the viability of cumulus cells also negatively influences oocyte meiotic maturation. We found that BOEC–Exo treatment to the primary cultured bovine cumulus cells significantly inhibited apoptosis rate as compared with the control cultured cells. 

The presence of metabolites in the culture medium were correlated with the developmental competence of embryos [42,43]. Several studies have identified that exosomes mediate metabolic reprogramming, and more specifically, lipid metabolism [26,27]. In our results, we found significant enhancement in GLUT1 in Exo-treated oocytes, which confirmed the previous findings that Exo improve the metabolism in the recipient cells (Figure 3) [26]. The direct relationship between lipid metabolism and exosomes has already been identified in several studies, and lipids significantly affect bovine embryo development [27,29]. So, we analyzed the expression of sirtuin 1 (Sirt1), which controls glucose and lipid metabolism, and we found it had a higher expression in the Exo-treated oocytes [44]. Furthermore, mitochondria play a fundamental role in glucose metabolism, and we found higher mitochondrial membrane potential in Exo-treated oocytes [45,46,47]. Moreover, we also examined lipid metabolism-related genes and found a higher expression of those genes in the Exo group (Figure 4). Consequently, the lipid concentration level was found to be significantly lower in the Exo-treated group as compared with the control group. PPARδ has recently been discovered to play a crucial function in embryo development and lipid metabolism [21,48]. We found significantly higher expressions and nuclear localization of the PPARδ protein in Exo-treated day-8 blastocysts. Embryo implantation potential is one of the most important predictive factors of successful pregnancy [49]. Several studies have used invasion assay to characterize the embryo implantation potential [21,50,51]. We also performed an invasion assay, assessed the embryo implantation and invasion potential, and identified that the 3% Exo group had a significantly high area of invasion.

## 5. Conclusions

In the present study, we explored the effects of BOEC–Exo on bovine oocyte maturation and early embryo developmental competence. We found that the addition of 3% Exo to IVM significantly improved oocyte meiotic maturation and subsequent embryonic development and competence. This research backs up the idea that modifying in vitro maturation conditions with exosomes produced from bovine oviductal epithelial cells has a good impact on the maturation of bovine oocytes. 

## Figures and Tables

**Table 1:** Primer sequences.

Gene Name	Primer Sequences	Accession Number
GAPDH	F: TTCAACGGCACAGTCAAGG R: ACATACTCAGCACCAGCATCAC	NM_001034034
GREM1	F: CAGTGCAACTCCTTCTACATCC R: GAGTTCAGGACAGTTGAGAGTGAC	XM_024997728.1
HAS2	F: TCTCTAGAAACCCCCATTAAGTTG R: ATCTTCCGAGTTTCCATCTATGAC	NM_174079.3
PTX3	F: CACAGGTCATGTTGTTCCTGAG R: CAGATATTGAAGCCTGTGAGTCTG	NM_001076259.2
GDF9	F: TGTTTAACCTGGATCGTGTTACTG R: AAACTCTGGCTCTTTTATCACCAG	NM_174681.2
CX43	F: CTTTCGTTGTAACACTCAACAACC R: GTAGAACACATGAGCCAGGTACAG	J05535.1
CX37	F: AGCCCGTGTTTGTGTGCCAG R: ACCAGGGAGATGAGTCCGACCA	NM_001083738.1
GLUT1	F: ATCCTCATTGCCGTGGTGCT R: ACGATGCCAGAGCCGATGGT	M60448
GLUT3	F: CGCCTTTGGCACTCTCAAC R: GCACTGGATGATGGCTGGTAA	AF308829
PGK1	F: CCTGTGGGTGTATTTGAATGG R: AGCACTTTACCTTCCAGGAG	BT021601.1
CPT1	F: GAGGGAGACTTTACACGGATGA R: AGATGTATTCCTCCCACCAGTC	NM_001304989
FABP3	F: GACAGCAAGAATTTCGATGACTAC R: CTGATCTCTGTGTTCTTGAAGGTG	NM_174313.2
PPARγ	F: ATATTTCCCTCTTTGTGGCTGC R: ATGGTTGTTCTGTAGGTGGAGT	XM_0249913
CPEB1	F: GTGTGGAGTGGCCTGGTAAG R: GAGAGCAAGCCTGAAGCAAG	XM_864691
SLC2A1	F: CCCCAGAAGGTGATTGAAGA R: GCCGAAACGGTTAACAAAAA	NM_174602.2
CD36	F: ACTGAGGATGACACGTTCA R: AATGGATCCGTATAGCCC	NM_174010
ATGL	F: CTGCTGACCACACTCTCCAA R: GGCGCGTATCATCAGGTACT	FJ798978.1

Abbreviations: F, forward; R, reverse.

**Table 2:** Bovine blastocysts developed from oocytes treated with Exo and control groups.

Groups	Oocytes, *n*	Presumed Zygotes, *n*	≥8-cell Embryos, n %	Total Blastocysts, *n %*	Hatched Blastocysts, *n %*
Control	314	294	164 (55.8 ± 3.40) ^b^	83 (28.2± 1.08) ^b^	18 (20.5 ± 4.24) ^a^
3% Exo	312	288	191 (66.3 ± 2.90) ^a^	104 (36.1 ± 0.67) ^a^	32 (27.9 ± 3.83) ^a^

^a,b^ Values with different superscripts in the same column are significantly different (*p* < 0.05).

## Data Availability

The data presented in this study are available on request from the corresponding author.
